# Viral Rebound Kinetics Correlate with Distinct HIV Antibody Features

**DOI:** 10.1128/mBio.00170-21

**Published:** 2021-03-09

**Authors:** Yannic C. Bartsch, Carolin Loos, Evan Rossignol, Jesse M. Fajnzylber, Dansu Yuan, Anchalee Avihingsanon, Sasiwimol Ubolyam, Thidarat Jupimai, Bernard Hirschel, Jintanat Ananworanich, Douglas A. Lauffenburger, Jonathan Z. Li, Galit Alter, Boris Julg

**Affiliations:** a Ragon Institute of MGH, MIT and Harvard, Cambridge, Massachusetts, USA; b Department of Biological Engineering, Massachusetts Institute of Technology, Cambridge, Massachusetts, USA; c Brigham and Women’s Hospital, Harvard Medical School, Boston, Massachusetts, USA; d HIV-NAT, The Thai Red Cross AIDS Research Centre, Bangkok, Thailand; e Tuberculosis Research Unit, Faculty of Medicine, Chulalongkorn University, Bangkok, Thailand; f Center of Excellence for Pediatric Infectious Diseases and Vaccines, Faculty of Medicine, Chulalongkorn University, Bangkok, Thailand; g Division of Infectious Diseases, Geneva University Hospital, Geneva, Switzerland; h Department of Global Health, University of Amsterdam, Amsterdam, The Netherlands; University of KwaZulu-Natal

**Keywords:** immune activation, viral reservoir, antibody function, glycosylation, human immunodeficiency virus

## Abstract

Plasma viremia reoccurs in most HIV-infected individuals once antiretroviral therapy (ART) is interrupted. The kinetics of viral rebound, specifically the time until plasma virus becomes detectable, differ quite substantially between individuals, and associations with virological and immunological factors have been suggested. Standard clinical measures, like CD4 T-cell counts and plasma HIV RNA levels, however, are poor predictive markers. Antibody features, including Fc functionality and Fc glycosylation have been identified as sensitive surrogates for disease activity in multiple diseases. Here, we analyzed HIV-specific antibody quantities and qualitative differences like antibody-mediated functions, Fc gamma receptor (FcγR) binding, and IgG Fc glycosylation as well as cytokine profiles and cellular HIV DNA and RNA levels in 23 ART-suppressed individuals prior to undergoing an analytical ART interruption (ATI). We found that antibodies with distinct functional properties and Fc glycan signatures separated individuals into early and delayed viral rebounders (≤4 weeks versus >4 weeks) and tracked with levels of inflammatory cytokines and transcriptional activity of the viral reservoir. Specifically, individuals with early viral rebound exhibited higher levels of total HIV-specific IgGs carrying inflammatory Fc glycans, while delayed rebounders showed an enrichment of highly functional antibodies. Overall, only four features, including enhanced antibody-mediated NK cell activation in delayed rebounders, were necessary to discriminate the groups. These data suggest that antibody features can be used as sensitive indicators of HIV disease activity and could be included in future ATI studies.

## Abstract (precis)

Plasma viremia reoccurs in most HIV-infected individuals once antiretroviral therapy is interrupted, and interindividual differences in the kinetics of viral rebound have been associated with virological and immunological factors. Antibody features, including Fc functionality and Fc glycosylation, have been identified as sensitive surrogates for disease activity in multiple diseases.

## INTRODUCTION

Human immunodeficiency virus (HIV) infection can be successfully controlled by modern antiretroviral therapy (ART). Despite the effective viral suppression below detectable limits, plasma viremia reoccurs in most individuals once ART is stopped. Nevertheless, the kinetics of viral rebound, specifically the time until plasma virus becomes detectable, differs quite substantially between individuals, and multiple virological and immunological factors have been proposed that might affect this process (1). Viral rebound is primarily fueled by the cellular HIV reservoir that is established early in infection, which persists in a latent state and is largely unresponsive to ART (2–4). Although the mechanisms of HIV persistence are incompletely understood, it has been suggested that continuous replenishment of the reservoir occurs through low-level ongoing virus production, perhaps in tissue compartments where ART penetration or activity is suboptimal. In contrast, viral replication or antigenic expression has been associated with chronic immune activation and inflammation, which is insufficiently reduced by ART (5). Furthermore, immune activation by itself can promote HIV transcription and virus production (5) and supports HIV persistence by inducing continuous proliferation of latently infected cells (6). The complexity of these relationships has made it difficult to measure/quantify the overall inflammatory state and the level of activation of the viral reservoir in individuals on ART, both of which are likely to be insufficiently captured in the usual clinical measurements of CD4 T-cell counts and plasma HIV RNA levels.

For decades, immunoglobulins have been used as serological markers for the diagnosis of infections but also for the assessment of immunity following infection or vaccination. Simple quantification of pathogen-specific IgG and IgM levels, however, does not adequately reflect the entirety of the antibodiome and misses many potential critical attributes that antibodies can provide in their role as sensitive sensors of human disease processes. Antibodies exist in many flavors and in addition to neutralization, can mediate a multitude of functions via their variable fragment crystallizable region (Fc region), including recruitment and activation of (innate) effector cells via the engagement of Fc gamma receptors (FcγR) on the surfaces of these cells. Functions like antibody-dependent cell-mediated cytotoxicity (ADCC), antibody-dependent cellular phagocytosis (ADCP), complement-dependent cytotoxicity (CDC), and anti-inflammatory activities have been proposed to play a role in HIV control (7, 8). Indeed, polyfunctional antibodies are enriched in HIV elite controllers (ECs) who can maintain undetectable viral loads in the absence of ART (9) and for whom reduced reservoir sizes have been described (10). Antibody characteristics, rather than titers alone, have also been linked with vaccine efficacy in HIV vaccine studies (11). Furthermore, the composition of glycans in the IgG’s Fc region can fine-tune functional and FcγR binding properties. These alterations alone have been identified as biomarkers for multiple diseases, including cardiovascular disease, inflammatory bowel disease, and diabetes (12–15) and more recently, specific IgG glycan signatures before ART interruption (ATI) have been associated as predictors for time to viral rebound (16).

Given the potential of antibody features to serve as a surrogate for disease activity, we applied a comprehensive antibody profiling approach to determine the landscape of the HIV humoral immune response in 23 ART-suppressed individuals prior to undergoing an ATI. In order to advance our understanding of non-clade B infection, we focused on individuals with CRF01-AE infection. We analyzed relative antibody quantities as well as qualitative differences like antibody-mediated functions, FcγR binding, and IgG glycosylation in an HIV-specific manner. We found that antibodies with distinct functional properties and Fc glycan signatures prior to ATI separated individuals with regard to time to viral rebound and tracked with the level of general inflammation and transcriptional activity of the viral reservoir. Specifically, only four selected features (increased anti-gp120 antibody induced CD107a, gamma interferon [IFN-γ], and macrophage inflammatory protein 1β (MIP-1β) expression in NK cells and increased engagement of FcγR2a of gp140-specific IgG in delayed rebounders) were necessary to discriminate the groups. A similar relationship of antibody profiles and viral transcriptional activity was observed in an additional cohort of HIV controllers where the same antibody features also separated individuals with undetectable HIV RNA from individuals with detectable but low HIV RNA levels. These data therefore suggest that antibody features can be used as sensitive indicators of HIV disease activity.

## RESULTS

### Cohort.

**FIG 1:**
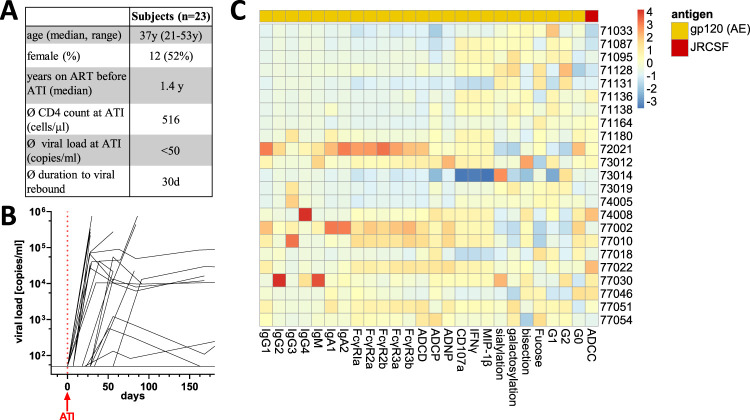
Comprehensive systems serology data of the tested cohort. (A) Characteristics of 23 HIV patients on ART with no detectable blood HIV levels (<50 copies/μl) and a CD4 count of more than 350 cells/μl in blood who underwent analytical treatment interruption (ATI). y, years; d, days. (B) Viral blood loads over the course of treatment interruption for individual patients. (C) Heatmap of Z-scored data of the antibodiome data set (antibody titer, FcR binding, ADCP, ANDP, ADNKA, ADCC, Fc glycosylation) for a representative antigen in HIV-positive (HIV+) individuals before treatment interruption.

We investigated HIV-specific antibody profiles in plasma from 23 ART-suppressed HIV-infected individuals, who had been enrolled in Thailand as part of the STACCATO trial, a CD4 T-cell count-guided analytical antiretroviral treatment interruption study (17). Our intention was to define the baseline landscape of HIV-specific humoral responses in ART-suppressed individuals prior to ATI. Patients were between 21 and 57 years old and had a CD4 T-cell count of >350 cells/μl (average, 516 cells/μl). Study participants were a median of 2 (interquartile range [IQR], 1 to 4) years infected and 1.46 (IQR 0.59 to 2.93) years on ART prior to the ATI (see Table S1 in the supplemental material). Initial ART was started in the chronic phase of infection in all included subjects. During ATI, viral load was measured every 4 weeks over the first 12 weeks and every 12 weeks thereafter to assess for viral rebound (defined as >50 copies [cp]/ml). Within the 23 individuals in this cohort, a wide distribution of time to viral rebound was observed, ranging from 1 to 120 weeks after the start of ATI. Viral rebound occurred within the first 4 weeks for most of the patients (mean time to viral rebound was 30 days), which is in line with other historically observed rebound times (1). Duration of ART prior to ATI did not correlate with time to viral rebound (Spearman *r *= 0.2, *P* = 0.3). All patients resumed ART per study protocol when the CD4 count dropped below 350 cells/μl (Fig. 1A and B).

10.1128/mBio.00170-21.1 TABLE S1 Clinical characteristics of the 23 selected STACCATO study participants. Download Table S1, PDF file, 0.05 MB. Copyright © 2021 Bartsch et al. 2021 Bartsch et al. https://creativecommons.org/licenses/by/4.0/ This content is distributed under the terms of the Creative Commons Attribution 4.0 International license.

### Antibody specificities.

To profile the HIV-specific antibody profile in this cohort, we screened plasma Ig titers against different HIV-1 proteins across different HIV-1 strains and clades. As expected, the highest titers were observed for HIV-1 gp120 and gp140 from the clade AE (CRF01), which is the dominant circulating HIV-1 clade in Thailand (18) (see Fig. S1 and S2 in the supplemental material). Titers for HIV envelope (Env) antigens from different strains within the same clade were highly correlated with each other, indicating an overall good recognition of the used antigens by plasma antibodies of the study population. Antibody responses across clade A and B Env antigens (gp120) were observed, suggesting recognition breadth. Titers for non-Env antigens, besides some detectable levels for anti-p24 antibodies, were below or in the range of the assay background. On the basis of the observed binding patterns, we expected comparable functional profiles across the antigens with correlated titer data (Fig. S2). For example, binding titers to gp120 antigens from different clades (A, B, or AE) are highly correlated with each other. Hence, for the more in-depth antibody profiling, we selected a clade AE- and a clade A-specific gp120 antigen (strains A244 and BG505, respectively). Additionally, we included a clade AE-derived gp140 antigen to detect responses against gp41.

10.1128/mBio.00170-21.3 FIG S1 Relative binding titers to multiple different HIV antigens at a fixed dilution were determined by Luminex. The heatmaps show individual values per subject and antigen for each determined antibody isotype/subclass. Plasma was collected before ATI and diluted 1:270 for IgG1 and 1:30 for all other titer determinations. Download FIG S1, PDF file, 0.2 MB. Copyright © 2021 Bartsch et al. 2021 Bartsch et al. https://creativecommons.org/licenses/by/4.0/ This content is distributed under the terms of the Creative Commons Attribution 4.0 International license.

10.1128/mBio.00170-21.4 FIG S2 Antibody titers shown in Fig. S1 were correlated (Pearson correlation) with each other according to subclass and isotype. The size of the circle indicates significance levels (larger diameter means lower *P* value; nonsignificant correlations are also shown). Color scale indicates positive or negative R values. Download FIG S2, PDF file, 0.4 MB. Copyright © 2021 Bartsch et al. 2021 Bartsch et al. https://creativecommons.org/licenses/by/4.0/ This content is distributed under the terms of the Creative Commons Attribution 4.0 International license.

### System serology.

To obtain an unbiased look at the antibody profiles in our study population, we applied a system serology approach to quantify 62 different binding or Fc functional antibody characteristics (Fig. S3). Subclass and isotype titers and the antigen-specific binding potential to different Fc gamma receptors (FcγR) were measured by Luminex multiplexing. We also systematically explored the potential of the patient’s antibodies to induce antibody-dependent complement deposition (ADCD), neutrophil phagocytosis (ADNP), monocyte phagocytosis (ADCP), and NK cell activation against the three selected antigens. Furthermore, we measured the NK cell-mediated antibody-dependent cytotoxicity (ADCC) to HIV-1-infected lymphocytes as well as the Fc glycosylation profile of gp120 (clade AE)-specific IgG antibodies. Overall, the antibody features were quite heterogeneous across individuals (Fig. 1C and Fig. S4). However, a clear correlation of features across antigens was observed (Fig. S4).

10.1128/mBio.00170-21.5 FIG S3 Overview of assays performed (left table) and antigens used (right table) under the systems serology approach. Download FIG S3, PDF file, 0.03 MB. Copyright © 2021 Bartsch et al. 2021 Bartsch et al. https://creativecommons.org/licenses/by/4.0/ This content is distributed under the terms of the Creative Commons Attribution 4.0 International license.

10.1128/mBio.00170-21.6 FIG S4 Pearson correlation of the systems serology data (Fig. 1C) by antibody feature (A) or individual (B). Only significant correlations are shown (*P* < 0.05), with red-yellow tiles indicating positive and green-blue tiles indicating negative correlations. Download FIG S4, PDF file, 0.1 MB. Copyright © 2021 Bartsch et al. 2021 Bartsch et al. https://creativecommons.org/licenses/by/4.0/ This content is distributed under the terms of the Creative Commons Attribution 4.0 International license.

**FIG 2:**
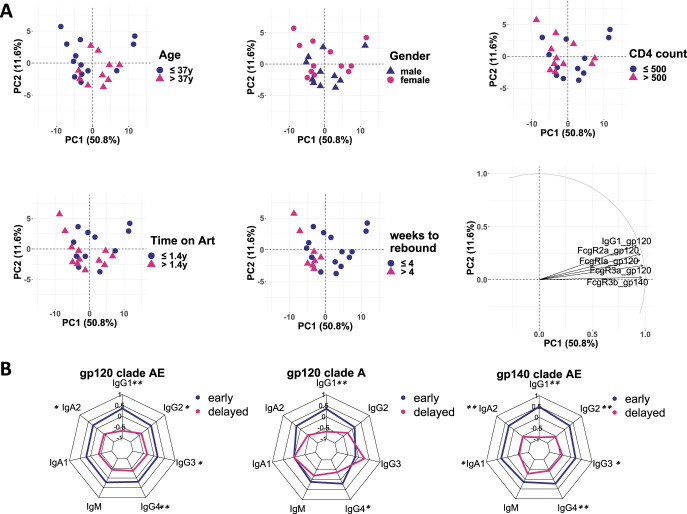
High HIV-specific antibody titer and FcγR binding are associated with early HIV rebound after analytical treatment interruption. (A) Principal-component analysis (PCA) of the generated antibody profile data set of HIV-specific serum antibodies before ATI. Each dot represents the value for one individual (*n* = 23) and was color coded by age (upper left panel), sex (upper middle panel), CD4 count (upper right panel), time on ART before ATI (lower left panel) or weeks to viral rebound (lower middle panel). The top five factors contributing to the distribution along PC1 and PC2 are shown in the loadings plot (lower right panel). (B) Radar plot of the average (after Z-scoring) antigen-specific antibody titers for the three HIV antigens for HIV+ individuals with early (blue; *n* = 15) or delayed (red; *n* = 8) viral rebound, respectively. Asterisks indicate statistically significant difference between the group in the univariate test after correction for multiple comparison (*, *P* ≤ 0.05; **, *P* ≤ 0.01).

In order to determine which factors could be involved in explaining antibody profile differences, we applied a nonsupervised principal-component analysis (PCA) to the entire data set. Fifty-one percent of the variation in the data was explained by principal component 1 (PC1). To probe for other confounders in our cohort that might explain the observed difference before ATI, we labeled the data points in our PCA analysis by demographic and clinical data. Neither age, sex, CD4 cell count at ATI, nor time on ART before ATI accounted for variation or clustering in the PCA. However, when we focused on time to viral rebound, we observed a distinct separation along PC1. Interestingly, among the top five contributing factors for the separation in PC1, gp120-specific antibody titer differences dominated (Fig. 2A). Hence, we compared the individual titers in early (≤4 weeks to rebound) or delayed (>4 weeks) rebounders. We observed significantly elevated levels of clade AE gp120- and gp140-specific titers for all four IgG subclasses and IgA2 in plasma from the early rebounders. Additionally, anti-gp140 IgA1 titers were also greater in this group. IgM titers were not significantly different between the groups. Counterintuitively, these data suggest that high HIV-specific IgG titers at the time of ATI are not protective against viral rebound but instead are associated with early viral rebound upon ATI (Fig. 2B, Fig. S5).

10.1128/mBio.00170-21.7 FIG S5 Plasma antibody titer of antibodies specific for gp120 clade AE (A), gp120 clade A (B), and gp140 clade AE (C) were determined by Luminex. Plasma was collected before ATI and diluted 1:270 for IgG1 and 1:30 for all other titer determinations. Data are also shown as averaged Z-scored values in the radar plots in Fig. 2B. Download FIG S5, PDF file, 0.5 MB. Copyright © 2021 Bartsch et al. 2021 Bartsch et al. https://creativecommons.org/licenses/by/4.0/ This content is distributed under the terms of the Creative Commons Attribution 4.0 International license.

### Increased Fc function is associated with delayed viral rebound.

**FIG 3:**
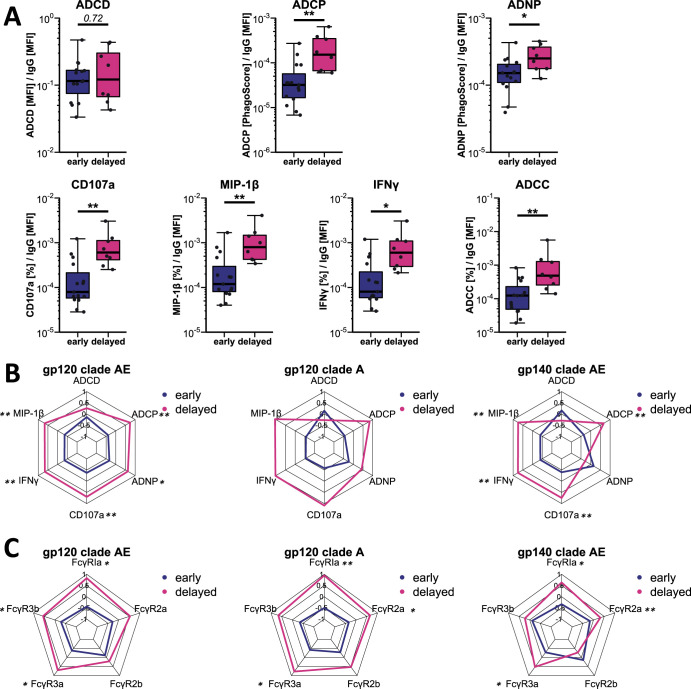
Patients with delayed viral rebound have augmented HIV-specific IgG Fc effector functions prior to ATI. (A) Antibody functions for gp120 clade AE-specific ADCD, ADCP, ADNP, and ADNKA as well as ADCC of JRCSF HIV-1-infected CEM cells before ATI were corrected for the individual’s gp120-specific total IgG titer (as determined by Luminex). MFI, mean fluorescence intensity. (B and C) Radar plot representation of the titer-corrected and Z-scored antibody functions (B) and FcγR binding (C) for each of the tested antigens (compare to panel A for gp120 clade AE). Asterisks indicate statistically significant differences between the group (early [*n* = 15] and delayed [*n* = 8]) in the univariate test after correction for multiple comparison (*, *P* ≤ 0.05; **, *P* ≤ 0.01).

We next compared antibody functionality between both groups (early (≤4 weeks to rebound) or delayed (>4 weeks to rebound)), focusing on ADCD, ADNP, ADCP, and NK cell activation. After correcting for antibody titers, we observed a significantly increased activation of NK cells in individuals that experienced a delayed rebound compared to early rebounders (assessed by expression of surface CD107, MIP-1β, and IFN-γ) against all three tested antigens. This increased NK activity also tracked with significantly augmented NK-mediated killing (ADCC) activity of HIV-infected CEM cells in the slow rebounders (adjusted *P* ≤ 0.01). Delayed rebounders also showed an increased potential to induce monocyte (THP-1 cell line) phagocytosis of immune complexes across the tested antigens (adjusted *P* ≤ 0.01). Similarly, neutrophil phagocytosis was elevated for clade AE gp120-specific immune complexes (adjusted *P* ≤ 0.05). No difference in the potential to activate complement was observed against the tested antigens. Taken together, these data indicate that while early rebounders had higher titers, the antibody Fc functional quality was inferior compared to delayed rebounders with regard to NK cell activity and monocyte phagocytosis (Fig. 3A and B and Fig. S6). While we observed a clear association with time to viral rebound, no specific functions were correlated with time to ART resumption (triggered by a drop in CD4 count below 350 cells/μl as per STACCATO protocol) or the ability to maintain viral loads below 10,000 copies/ml during ATI for a longer period of time (data no shown).

10.1128/mBio.00170-21.8 FIG S6 (A and B) Antibody functions for gp120 clade A (A)- and gp140 clade AE (B)-specific ADCD, ADCP, ADNP, and ADNKA before ATI were corrected for the individual’s gp120 clade A- or gp140 clade AE-specific total IgG titer (as determined by Luminex), respectively. Data are shown as averaged Z-scored values in the radar plots in Fig. 3B. gp120 clade AE (C)-, gp120 clade A (D)-, and gp140 clade AE (E)-specific binding of plasma antibodies to FcγR in HIV+ before ATI were corrected for the individual’s antigen-specific total IgG titer (as determined by Luminex), respectively. Data are shown as averaged Z-scored values in the radar plots in Fig. 3C. Download FIG S6, PDF file, 0.7 MB. Copyright © 2021 Bartsch et al. 2021 Bartsch et al. https://creativecommons.org/licenses/by/4.0/ This content is distributed under the terms of the Creative Commons Attribution 4.0 International license.

To determine whether the observed Fc functional differences were caused by changes in FcγR binding, we compared the titer-normalized FcγR binding characteristics in the two groups (Fig. 3C and Fig. S6). Intriguingly, delayed rebounders exhibited significantly increased binding to FcγRI and FcγRIIIa across all tested antigens. This might explain the observed augmented phagocytosis and, in particular for FcγRIIIa, the enhanced NK cell activation and cytotoxicity (ADCC) mediated by antibodies in the delayed rebounder group. Binding to FcγRIIa and FcγRIIIb was also augmented in delayed rebounders, though it did not reach statistical significance for all antigens. Interestingly, no difference was observed for binding to FcγRIIb, the only inhibitory Fcγ receptor. Together, these data suggest that the humoral response in early and delayed rebounders differs in the quality of Fcγ receptor binding and subsequently, antibody-mediated functions.

### Proinflammatory IgG Fc glycans in patients with early viral rebound.

**FIG 4:**
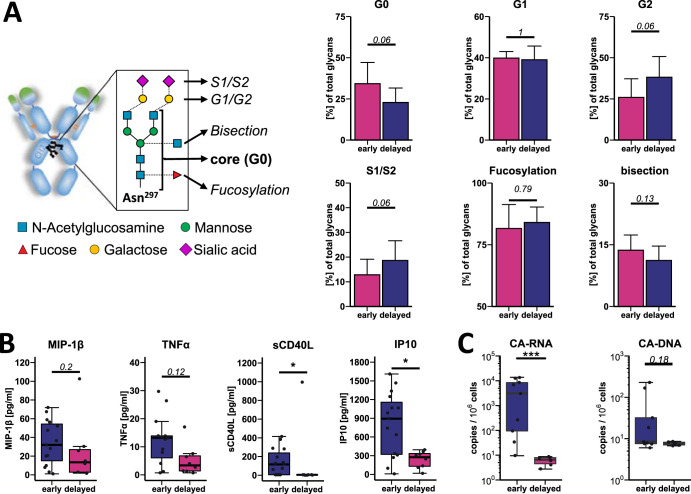
Early viral rebounders show signs of augmented immune activation and have a larger viral reservoir size prior to ATI. (A) The conserved IgG Fc glycan at Asn297 in the CH2 domain is a biantennary complex-type glycan with a chiotobiose core glycan. Fucosylation at the first *N*-acetylglycosamine, a bisecting *N*-acetylglucosamine (GlcNAc) at the branching mannose or elongation of the one or both antenna with galactose (G1 or G2) and sialic acid (S1 or S2) are commonly observed modifications. Fc glycosylation of gp120 (clade AE)-specific IgG before ATI was analyzed in patients with early (≤4 weeks; *n* = 15) and delayed (>4 weeks; *n* = 8) viral rebound by capillary electrophoresis. The frequency of nongalactosylated/sialylated glycans (G0), mono- and bigalactosylated (nonsialylated) glycans (G1 and G2, respectively) and mono- and bi-sialylated glycans combined (S1/S2), fucosylation and bisecting GlcNAc are shown, respectively. (B) Plasma cytokine concentration was measured by multiplexing. Shown are the concentrations (in picograms per milliliter) of MIP-1β, TNF-α, sCD40L, and IP-10 before ATI in patients with early (≤4 weeks; *n* = 15) and delayed (>4 weeks; *n* = 8) viral rebound. (C) HIV reservoir was estimated by cell-associated (CA) RNA and CA-DNA and measured by real-time PCR in PBMCs of patients with early (≤4 weeks, *n* = 9) and delayed (>4 weeks, *n* = 6) viral rebound. PBMC samples were taken before ATI, and CA-RNA and CA-DNA levels were normalized to copies per million cells based on CCR5 quantification. The calculated limit of detection is used for samples with values below this threshold.

IgG binding to Fcγ receptors and subsequently IgG-mediated functions are influenced by both subclass selection, which did not differ substantially between the groups, and the composition of the Fc N-linked glycan that is attached to the CH2 domain of all IgGs. For example, fucosylation of the IgG1 core glycan decreases FcγRIIIa binding and subsequently reduces NK cell-mediated killing (19). The role of the addition of galactose and sialic acid to the glycan antenna with regard to FcγR binding is less well characterized. Hence, we investigated the Fc glycosylation pattern of gp120-specific IgGs. In our cohort, we did not observe significant differences in the degree of fucosylation between early and delayed rebounders; however, patients with early viral relapse tended to have less galactosylated (more G0 structures, early versus delay rebounder, 33.98% versus 22.80% of all detected glycans, adjusted *P* = 0.06) and sialylated IgG Fc glycans (early versus delayed rebounders, 13.65% versus 18.63% of all detected glycans, adjusted *P* = 0.06). This is consistent with a recent study that found higher pre-ATI levels of the IgG glycan G2, to be significantly associated with a longer time to viral rebound (16). Indeed, decreased levels of IgG Fc galactosylation and sialylation, as they were observed for early rebounders here, are associated with inflammation and augmented immune activation in the contexts of autoimmune syndromes, cancer, and infections (20–23). These findings therefore suggest that the observed glycan pattern in early HIV rebounders point to an increased immune activation state, potentially in the setting of an overall prominent inflammatory milieu (Fig. 4A).

### Inflammatory cytokine milieu in early rebounders.

To further evaluate a potentially increased inflammatory state in our cohort, we analyzed plasma levels of 15 cytokines by multiplexing. Indeed, soluble CD40 ligand (sCD40L) and interferon-inducible protein of 10 kDa (IP-10) which have both been previously associated with untreated HIV infection and have been associated with disease progression (24–26) were significantly upregulated (sCD40L, 140.3 ± 137.2 pg/ml versus 3.4 ± 2.0 pg/ml; adjusted *P* value = 0.039; IP-10, 805.1 ± 493.973 pg/ml versus 228.2 ± 132.2 pg/ml, adjusted *P* value = 0.039, mean levels ± standard deviations [SD]) in patients with a viral rebound of ≤4 weeks. While the proinflammatory cytokines MIP-1β and tumor necrosis factor alpha (TNF-α) trended toward higher levels in this population, the difference did not reach statistical significance (TNF-α, 11.9 ± 8.3 pg/ml versus 5.1 ± 5.1 pg/ml; adjusted *P* value = 0.12; MIP-1β, 34.3 ± 23.0 pg/ml versus 24.1 ± 31.4 pg/ml; adjusted *P* value = 0.195, mean levels ± standard deviations [SD]). Levels for epidermal growth factor (EGF), Eotaxin, FDF-2, Gro, interleukin 1 receptor antagonist (IL-1RA), interleukin 3 (IL-3), IL-5, IL-8, MCP-1, macrophage-derived chemokine (MDC), and vascular endothelial growth factor (VEGF) did not differ between the groups (data not shown). These data suggest that consistent with the “inflamed” Fc glycosylation signatures, early viral rebounders appear to experience elevated levels of immune activation before treatment interruption (Fig. 4B).

### Reduced CA-DNA and RNA in delayed rebounders.

The size of the latent HIV reservoir has been correlated with the time to viral rebound in prior studies. Specifically, increased cell-associated DNA (CA-DNA) was used to estimate the reservoir size and negatively correlated with viral rebound times (27, 28). However, high viral transcriptional activity of latent cells (CA-RNA) was a predictive marker for early viral rebound in another study (29–31). Low-level viral replication, i.e., in solid tissues, despite plasma HIV RNA levels that are below the clinical test detection limits (i.e., <50 cp/ml) have been reported, but the activity of this low-level viral replication cannot easily be measured but might have a significant impact on viral kinetics during ATI (32, 33). We therefore quantified the cell (peripheral blood mononuclear cell [PBMC])-associated HIV DNA (CA-DNA) and HIV RNA (CA-RNA) levels to estimate genomic integration and viral replication of the reservoir, respectively. Interestingly, integrated CA-DNA levels were below the detection limit for all patients (detection limit was calculated per sample) in the delayed rebounders but not the early rebounder group (on average 59.5 cp/10^6^ PBMCs in the early rebounders versus <7.6 cp/10^6^ PBMCs for delayed rebounders; adjusted *P* = 0.18), and CA-RNA was higher in early rebounders (on average, 5,024 cp/10^6^ PBMCs in the early rebounders versus 6 cp/10^6^ PBMCs in the delayed rebounders; adjusted *P* = 0.0008). These findings indicate a potentially enlarged and far more active reservoir in early rebounders, with an increased production of HIV antigens that could explain the observed increased inflammatory markers and also the increased total antibody titers with inflammatory Fc glycans (Fig. 4C).

### Antibody characteristics are HIV specific.

**FIG 5:**
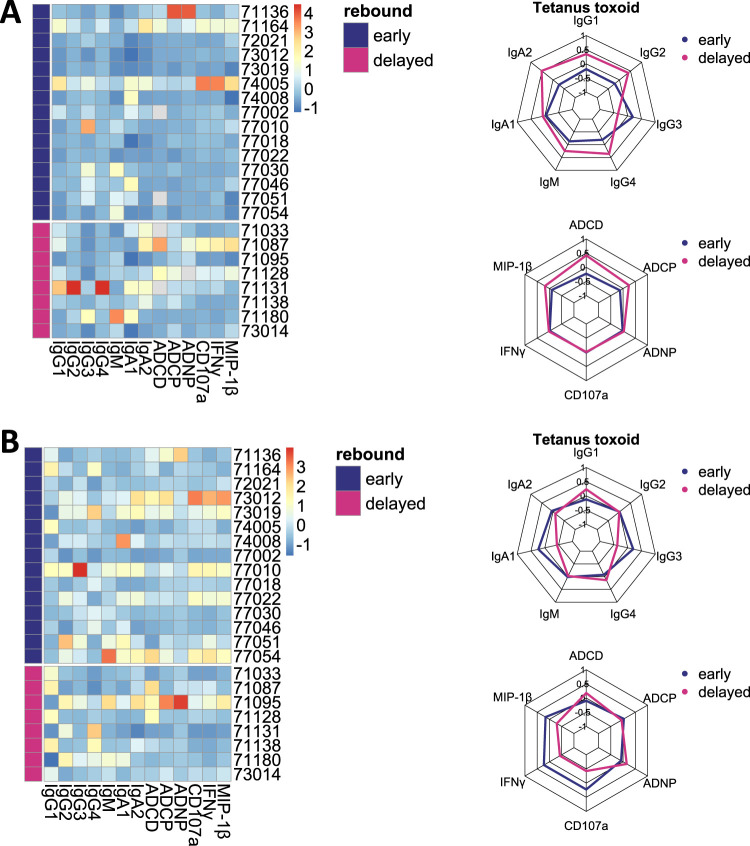
Tetanus-specific humoral immune responses are similar between early and delayed viral rebounders. Antibody titers (IgG1 to IgG4, IgM, IgA1 and IgA2) and functions (ADCD, ADCP, ADNP, and ADNKA) were determined against tetanus toxoid antigen. Data in panel A were not corrected, while the tetanus total IgG titer corrected data were used in panel B. The heatmaps (left panel) show the Z-scored data for each individual and measurement. The radar plots show averages for each measured titer (upper right) and functions (lower right) between early (≤4 weeks; *n* = 15) and delayed rebounders (>4 weeks; *n* = 8), respectively.

Given the substantial differences in HIV-specific antibody features, specifically with regard to Fc functionality and FcγR binding, between individuals with early or delayed viral rebound, we were interested in determining whether the observed variation was the result of a superior host immune response versus an antigen-specific process. We therefore measured characteristics of antibodies directed toward an unrelated antigen, namely, Clostridium tetani toxoid, using plasma from the same time point as had been used for the HIV antibody profiling. Tetanus toxoid was chosen under the assumption that antibody responses were from vaccination and therefore more standardized across individuals. In contrast to the difference seen with HIV, neither tetanus-specific antibody isotype and IgG subclass titers nor functional profiles, including ADCD, ADCP, ADNP, and antibody-dependent NK cell activation (ADNKA) differed between early and delayed HIV rebounder (Fig. 5). These data therefore suggest that the observed differences in the antibody pattern were limited to HIV-specific immune responses and not caused by a systemic difference in the subject’s immunity.

### Minimal humoral immune correlates discriminate early and delayed rebounders.

**FIG 6:**
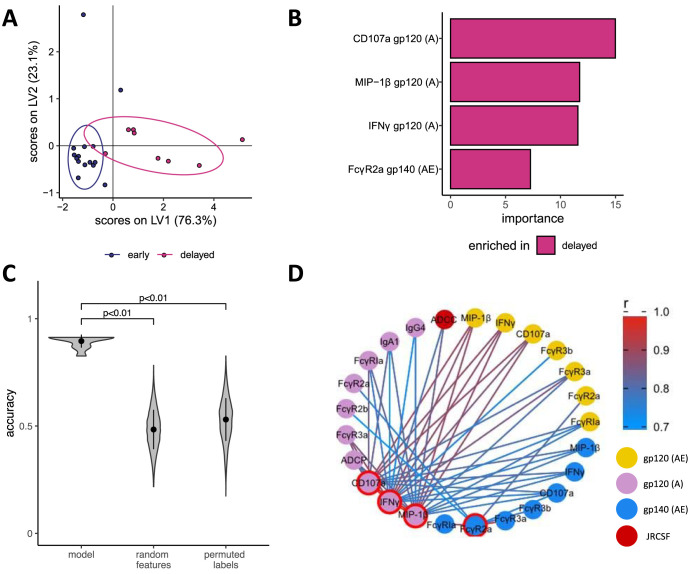
Computational machine learning model. (A) O-PLS-DA latent variable (LV) score plot for the features selected using a random forest recursive feature elimination approach to classify early and delayed rebounds. Ellipses show the distribution of the groups as 95% confidence levels assuming a multivariate Student’s *t* distribution. (B) Selected features are ranked according to their importance calculated as mean decrease in accuracy and colored according to their enrichment in early or delayed rebounders. (C) Violin plot for the accuracies of the random forest model from 10 repetitions of fivefold cross-validation for the actual model and models based on size-matched random features and randomly permuted labels. For the random feature and permuted label models, 100 random permutations are performed within each of the 10 repetitions. The *P* values are determined based on the probability that the accuracy of the permuted and random feature model is higher than for the actual model. (D) Cocorrelate network showing features that are significantly correlated with the selected features (red border) and have a Spearman rank correlation coefficient |*r*| > 0.7. Edges are colored according to their correlation coefficient, and significances were corrected for multiple hypothesis testing with the Benjamini-Hochberg procedure.

Given the association between humoral immune profiles and time to rebound following ATI, we applied a computational machine learning approach to identify minimal antibody features which discriminate early and delayed rebounders and, hence, might play a role in viral control but could also be developed further as a marker of reservoir activity. A random forest recursive feature elimination approach was used to select a minimal number of features necessary to differentiate between early and delayed responders. The robustness and significance of the model were then assessed by comparing the actual model against models based on fold-specific size-matched random features and randomly permuted labels within a fivefold cross-validation framework. For 20 repetitions of this procedure, the model achieved an average cross-validation accuracy of 89%. Strikingly, the increased NK cell activation and engagement of FcγR2a by gp140 (clade AE)-specific IgG in delayed rebounding patients was particularly important for the model, as these functions were among the top four selected features to discriminate early and delayed rebounders in the random forest model. In a cocorrelation network analysis showing significant correlation of the selected features, NK cell activation was highly correlated for all antigens and interestingly also to cellular phagocytosis (ADCP) (|*r*| > 0.7 and *P* < 0.05) (Fig. 6).

### Minimal immune features correlate with viral activity in spontaneous HIV controllers.

**FIG 7:**
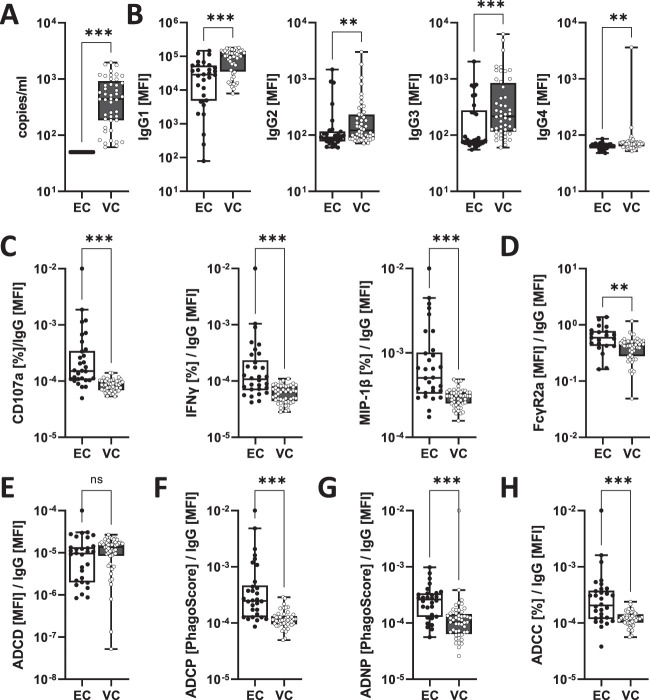
Antibody features track with disease activity in HIV controllers. HIV-gp120 (clade B: SF162) specific antibody profiles were analyzed in elite controllers (EC) (*n* = 30) and viremic controllers (VC) (*n* = 48). (A) Plasma viral loads in the absence of treatment were used to define EC (<50 copies [cp]/ml) and VC (50 to 2,000 cp/ml). (B) IgG antibody titer to gp120 (clade B; SF162). (C to H) IgG-normalized NK-mediated functions (C), FcγR2a binding (D), ADCD (E), ADCP (F), ADNP to gp120 (G), and ADCC to gp120 (H) pulsed CEM-NKr cells was analyzed. Asterisks indicate statistically significant differences between the groups after correction for multiple comparison (*, *P* ≤ 0.05; **, *P* ≤ 0.01; ***, *P* ≤ 0.001; ns, not significant). The cohort and data shown in this figure have been described before (9).

While only four features were sufficient to discriminate between early and delayed rebounders and identified individuals with increased viral/disease activity, we thought to investigate whether similar differences exist in a different cohort of individuals living with HIV. We selected HIV controllers who have distinctly different viral activity in the presence of preserved immune functionality, namely, elite controllers (ECs) with undetectable HIV RNA levels and viremic controllers (VCs) with detectable but low levels (<2,000 cp/ml). Along these lines, VCs are reported to have larger reservoir size (CA-DNA) than ECs (10), an observation that at least relatively resembles our findings of CA-DNA levels in early and delayed rebounders. In order to replicate our analysis, we utilized and reanalyzed systems serology data of HIV controllers that was previously published (9). As per cohort definition, viral loads were undetectable in elite controllers but clearly present in viremic controllers in this data set, indicating higher disease/viral activity in viremic controllers (Fig. 7A).

In line with our observation in early rebounders that had evidence of increased viral activity while on ART, viremic controllers had significantly higher gp120-specific IgG1 titers compared to elite controllers supporting a higher immune activation in this patient subset (Fig. 7B). When we analyzed the four identified features, namely, CD107a, MIP-1β, and IFN-γ expression of NK cells and FcγR2a binding (Fig. 7C and D), that had tracked with delayed viral rebound and reduced disease activity at baseline in the treatment interruption cohort, we found that all features were significantly upregulated in elite controllers compared to viremic controllers. These data therefore confirm the relationship between specific antibody features and viral/disease activity. In addition and similar to the ATI cohort, we observed no significant difference in the complement-fixing activity between ECs and VCs but increased potential to induce phagocytosis in neutrophils and THP-1 monocytes and to mediate ADCC in ECs (Fig. 7E to G). Collectively, the findings in the additional cohort largely resemble the findings in the treatment interruption cohort: while VCs had higher viral loads and gp120-specific IgG1 titers, gp120-specific antibodies of ECs had higher potential to mediate ADCC, activate NK cells, neutrophils, and monocytes. These findings therefore support our conclusion that distinct antibody features track with viral disease activity.

## DISCUSSION

Here, we investigated the antibody profiles of 23 HIV-infected ART-suppressed individuals prior to treatment interruption using a comprehensive system serology approach. Using this unbiased approach, we identified two distinct groups based on antibody features alone. Although the groups did not differ in age or baseline CD4 counts, the time it took during ATI for detectable plasma HIV RNA to occur (≤4 weeks versus >4 weeks to rebound), separated them in a principal-component analysis. Antibody features furthermore tracked with inflammatory cytokine levels and cellular HIV transcriptional activity. These findings therefore suggest that humoral immune response signatures are sensitive indicators of disease activity and are able to distinguish between even small differences in viral rebound kinetics.

In our study, lower anti-Env titers were observed for the delayed rebounding group; however, the HIV-specific antibody functional quality was superior with an increased potential to mediate ADNP, ADCP, NK activation, and ADCC. Indeed, NK cell activation and ADCC have been associated with the elimination of infected (reservoir) cells, and ADCP can directly remove antibody-opsonized virions (23, 34, 35). While only four antibody features were necessary to build a robust model, which can correctly discriminate early and delayed rebounders, an increased potential to activate NK cells was almost sufficient to split the groups. NK cell activation was highly correlated across HIV antigens and also correlated with increased ADCP. Whether this increased antibody functionality in delayed rebounders actively contributed to a reduction in reservoir size and/or slowed down viremia is yet to be determined. Moreover, the potential role of antibody-mediated function in viral control should be evaluated in future ATI studies.

The enhanced antibody functionality in the delayed rebounders did not seem to be the result of an overall more functional humoral host response, as we did not detect any significant differences in the antibody quantity and quality toward tetanus within the two groups. One could argue that the tetanus response, as likely having been induced by vaccination, differs from a response induced during natural infection. Our data, however, instead suggest that despite clinical suppression of plasma viremia, persistent B-cell induction occurred primarily in the early rebounders, potentially due to ongoing antigenemia in the tissues, leading to higher antibody titers overall but with less functionality, similar to an exhausted phenotype in the setting of a more inflamed milieu.

Consistent with the hypothesis of ongoing viral replication, we observed significantly elevated CA-RNA levels in early rebounders and substantially lower CA-DNA levels in delayed rebounders. It is likely that augmented viral transcription (i.e., CA-RNA) also leads to increased translation of viral antigens (36). Such antigens may not result in the formation of mature viral particles, but they can lead to immune activation and higher HIV-specific antibody titers (37). In line, elevated markers for T-cell exhaustion were associated with larger reservoir size (CA-DNA) and have been proposed as predictive markers for early viral rebound time upon treatment interruption (38). CA-RNA and CA-DNA are only bulk approximations of the viral reservoir, and more accurate reservoir measurements like IPDA (intact proviral DNA assay) (39) could be more informative. Unfortunately, these assays are currently qualified only for clade B infection (personal communication from Robert Siliciano) and not yet available for clade CRF01-AE, the strain that the study patients in our cohort are infected with.

Factors like pre-ART viral loads, duration of ART, and even CD4 counts at ART initiation might impact the size of the viral reservoir, and consequently could affect the level of immune activation and antibody profiles. However, in a linear model, we did not observe any significant associations of ART duration and CD4 count prior to ART or CD4 counts at ATI initiation, nor associations with viral loads pre-ART initiation with the observed antibody features (see Fig. S7 in the supplemental material). Furthermore, the HIV reservoir establishes early after infection, and likewise, early initiation of ART can limit the reservoir size. We therefore thought to investigate whether we can find similar association between humoral antibody profile and viral relapse in 13 HIV-infected individuals who started ART during the acute phase of infection and then underwent ATI as part of the clinical trials RV397 and RV411 in Thailand (40, 41). Although all individuals were sampled at similar time points, before and during ATI, as the STACCATO participants, we were not able to detect considerable HIV-specific antibody (Ab) titers in these individuals (Fig. S8). Early ART initiation might have suppressed viral replication, and subsequently, an initial humoral immune response was either insufficiently primed or not sustained.

10.1128/mBio.00170-21.9 FIG S7 Effects of viral loads and CD4 counts prior to ATI and prior to first ART on antibody features. Linear models were built to assess the influence of ART duration prior to ATI (A), CD4 count prior to ATI (B), CD4 count prior to first ART (C), or viral load prior to first ART (D) in addition to time to viral rebound after ATI (Ab features the approximate time to viral rebound plus covariate). T-values (correlation coefficient divided by standard deviation [SD]) are shown on the *x* axis, and the negative logarithmic *P* values (after correction for multiple testing by the Benjamini-Hochberg approach) are shown on the *y* axis. Features above the horizontal line are considered to be significantly correlated (*P* < 0.05) with the covariate. Download FIG S7, PDF file, 0.09 MB. Copyright © 2021 Bartsch et al. 2021 Bartsch et al. https://creativecommons.org/licenses/by/4.0/ This content is distributed under the terms of the Creative Commons Attribution 4.0 International license.

10.1128/mBio.00170-21.10 FIG S8 Lack of HIV-specific immunoglobulins in patients who started antiretroviral therapy early in the acute phase of infection. gp120 (clade AE and clade B)-specific IgG1 (A) and IgM (B) responses were analyzed by Luminex in HIV-infected individuals who initiated ART in the acute phase of infection (*n* = 13 individuals from the RV397 and RV411 ATI studies; from the RV397 study, only placebo recipients were included) or in the chronic phase of infection (*n* = 23 individuals from the STACCATO study). Samples were collected directly prior to ATI in both cohorts in Thailand, and all study participants were fully viral suppressed at the time of ATI. Download FIG S8, PDF file, 0.05 MB. Copyright © 2021 Bartsch et al. 2021 Bartsch et al. https://creativecommons.org/licenses/by/4.0/ This content is distributed under the terms of the Creative Commons Attribution 4.0 International license.

In addition to apparently increased transcriptional activity, we detected indicators of a more inflammatory cytokine milieu in early rebounders, namely, elevated levels of IP-10, and soluble CD40L. IP-10 has been associated with disease progression and immune activation in HIV-1 infections (24, 25), and elevated IP-10 has also been associated with immunological treatment failure in HIV pointing to its potential to track HIV activity in ART-treated individuals (42). CD40L on T cells can stimulate B-cell activation and maturation during cognate interactions, and in the context of HIV, sCD40L was found to be gradually enhanced in the serum of HIV-infected individuals in the absence of ART (26).

Indeed, as another correlate of immune activation, we found less galactosylated and sialylated Fc glycans on gp120-specific IgG Abs in the early rebounders. These glycosylation patterns have been associated with enhanced immune activation in the contexts of autoimmune syndromes or chronic and acute infections (20–23), and Fc glycosylation profiles can serve as sensitive and pathogen-specific markers of disease activity. For example, Giron et al. recently reported bulk IgG glycomic signatures in two cohorts in the United States and in South Africa that informed post-ATI time-to-viral-rebound and viral setpoints (16). Similar differences in Fc galactosylation were seen between the early and delayed rebounders in our cohort from Thailand, suggesting that these markers are independent of geographical influence, including the HIV clade.

Interestingly, antibody features that tracked with viral transcriptional activity in chronically infected individuals on ART and consequently correlated with time to viral rebound following ATI also discriminated HIV controllers with undetectable HIV RNA levels from HIV controllers with detectable but low viral loads. While antibody titers were higher in VCs, titer corrected functions were increased in ECs. This observation therefore confirms the exquisite sensitivity of antibody features as marker of viral activity in a different cohort and although the observed antibody profiles in delayed rebounders and ECs were qualitatively similar, it remains to be determined whether a causal relationship between antibody functions and viral control following ATI exists.

Taken together, here we identified HIV-specific antibody profiles that clearly identified individuals with increased immune activation and cellular transcriptional activity and tracked with the time to viral rebound following ATI. Measuring these profiles therefore enabled a comprehensive look at disease activity that was not captured by levels of CD4 T cells and HIV RNA in plasma. Given the technical complexities of measuring the size of the viral reservoir or quantifying the levels of inflammation, in particular in nonblood tissue compartments (43), easy-to-access plasma antibodies combined with high-throughput system serology might be useful tools for classifying individuals based on HIV disease activity and could be included in future ATI studies.

## MATERIALS AND METHODS

### Study population and sample collection.

Samples were collected from 23 HIV-seropositive adults in Thailand before they underwent their first analytical treatment interruption (ATI) as part of the STACCATO trial. Individuals for this study were primarily selected depending on availability of plasma samples. STACCATO was a international multicenter trial, but given our focus on CRF01-AE infection, only individuals who participated in Thailand were selected. Before ATI, patients received a daily treatment for at least 6 months with two nucleoside reverse transcriptase inhibitors (NRTIs) plus saquinavir-ritonavir. Seven of 23 individuals (30.4%) had to previously (before study entry) change their therapy regimen once, due to reasons other than virological failure (i.e., toxicity, new treatment guidelines, etc). No participant reported a treatment interruption period prior to entry into the study. At ATI, viral loads were below detection limit and CD4 counts above 350 per μl.

The 13 individuals, who had initiated ART during the acute phase of the infection, were identified from the RV397 and RV411 studies conducted at the Thai Red Cross AIDS Research Centre in Bangkok with support by the U.S. Military HIV Research Program (MHRP). While the RV397 study included interventions with investigational products, only individuals in the placebo control group were included. The nature of these studies was to recruit participants during the hyperacute to acute phase (Fiebig I-II) of the infection. The individuals were sampled right before the ATI initiation similar to the individuals in the STACCATO study.

### Antigens and biotinylation.

Recombinant HIV Env gp120 (clade AE), gp120 (clade A) antigens were purchased from Immune Technology Corp. (NY, USA), tetanus toxoid from MassBiologics (MA, USA). Gp140 (clade AE) was obtained from Duke University DHVI Protein Production Facility. If required by the assay, antigens were biotinylated with *N*-hydroxysuccinimide (NHS)-Sulfo-LC-LC kit according to the manufacturer’s instruction (Thermo Fisher, MA, USA). Excessive biotin was removed by size exclusion chromatography using Zeba-Spin desalting columns (7-kDa cutoff; Thermo Fisher).

### Antibody quantification and FcR binding.

Relative antigen-specific antibody titers and Fc receptor binding were measured as described before (44). Nonbiotinylated antigens were carboxy coupled to Luminex microspheres (Luminex Corp., TX, USA) and incubated with different plasma dilutions (1:30, 1:90, or 1:270). Isotypes and subclasses were probed with a fluorophore-tagged secondary antibody. For Fc gamma receptor binding, the respective Fc gamma receptor was used instead of the secondary antibody.

### Antibody-dependent complement deposition (ADCD).

Complement deposition was performed as described before (45). In brief, biotinylated antigens were coupled to fluorescent neutravidin beads (Thermo Fisher) and incubated with 10 μl of 1:10 diluted plasma. Beads were incubated with guinea pig complement in GVB++ buffer (Boston BioProducts, MA, USA) for 20 min at 37°C. Deposited C3 on beads was stained with anti-guinea pig C3 antibody labeled with fluorescein isothiocyanate (FITC) (MP Biomedicals, CA, USA) and analyzed by flow cytometry on a LSRII instrument (BD Biosciences, CA, USA).

### Antibody-dependent neutrophil phagocytosis (ADNP).

Phagocytosis score of primary human neutrophils was determined as described before (46). Biotinylated antigens were coupled to fluorescent neutravidin beads (Thermo Fisher) and incubated with 50 μl of 1:200 diluted plasma. Primary cells were derived from ammonium-chloride-potassium (ACK) buffer-lysed whole blood from healthy donors and incubated with immune complexes for 1 h at 37°C. Neutrophils were stained for surface CD66b (Biolegend, CA, USA; clone G10F5) expression, fixed with 4% paraformaldehyde and analyzed by flow cytometry on a LSR Fortessa (BD).

### Antibody-dependent THP-1 cell phagocytosis (ADCP).

THP-1 phagocytosis assay was performed as described before (47). In brief, biotinylated antigens were coupled to fluorescent neutravidin beads (Thermo Fisher) and incubated with 50 μl of 1:200 diluted plasma. THP-1 monocytes were added to the beads, incubated for 16 h at 37°C, fixed with 4% paraformaldehyde, and analyzed by flow cytometry on a LSR Fortessa (BD).

### Antibody-dependent NK activation (ADNKA).

To determine antibody-dependent NK cell activation, enzyme-linked immunosorbent assay (ELISA) plates (Thermo Fisher) were coated with nonbiotinylated antigen and then blocked. One hundred microliters of a 1:50 sample dilution was added to each well. NK cells were isolated from unidentified buffy coats from healthy donors using the RosetteSep NK cell enrichment kit (STEMCELL Technologies, MA, USA) and stimulated with human recombinant IL-15 (rhIL-15) (1 ng/ml; STEMCELL Technologies) at 37°C overnight. NK cells were added to the ELISA plate and incubated together with anti-CD107a (BD, clone H4A3), brefeldin A (Sigma-Aldrich, MO, USA), and monensin (BD) for 5 h at 37°C. Next, cells were surface stained for CD56 (BD, clone B159), CD16 (BD, clone 3G8), and CD3 (BD, UCHT1). After fixation and permeabilization with FIX & PERM cell permeabilization kit (Thermo Fisher), cells were stained for intracellular markers MIP-1β (BD, clone D21-1351) and IFN-γ (BD, clone B27). NK cells were defined as CD3^−^ CD16^+^ CD56^+^ and frequencies of degranulated (CD107a^+^), gamma interferon-positive (IFN-γ^+^), and MIP1β^+^ NK cells determined by flow cytometry on a LSR Fortessa (BD) (48).

### Antibody-dependent cellular cytotoxicity (ADCC).

CEM-NKr CCR5^+^ cells (49–51) (NIH AIDS reagents program) were infected with vesicular stomatitis virus glycoprotein (VSV-g)-pseudotyped HIV-1 (strain JRCSF, 0.5 IU/cell). Infection was facilitated with Polybrene (4 μg/ml) and spinoculation (800 × *g* for 45 min at room temperature [RT]). Cells were then incubated for 2 days at 37°C and media (RPMI supplemented with 10% fetal bovine serum [FBS], l-glutamine, and penicillin/streptomycin [Pen/Strep]) exchanged once 6 h after infection. NK cells were isolated from buffy coats from healthy donors using the RosetteSep NK cell enrichment kit (STEMCELL Technologies) and stimulated with rhIL-15 (1 ng/ml; STEMCELL Technologies) at 37°C overnight. Infected CEM-NKr CCR5^+^ cells were labeled with CellTrace far red cell proliferation kit (Thermo Fisher) before 1:50 diluted plasma sample were added together with the NK cells. After 4 h of incubation at 37°C, cells were stained with LIVE/DEAD Fixable Blue Dead Cell Stain kit (Thermo Fisher). Cells were then fixed and permeabilized with Fixation/Permeabilization Solution kit (BD) and stained for intracellular HIV-p24 (Beckman Coulter, CA, USA; clone KC57). Cell killing was calculated as frequency of viable, p24^+^ CEM-NKr CCR5^+^ cells compared to a negative control without antibodies (phosphate-buffered saline [PBS]).

### Antigen-specific IgG Fc glycosylation.

Plasma samples were diluted 1:10 with PBS and incubated overnight at 4°C with biotinylated gp120 (clade AE) coupled to magnetic streptavidin beads (NEB, MA, USA). Unspecific antibodies were washed away with PBS, and IgG-Fc fragments were enzymatically generated using IdeZ (NEB). IgG-Fc glycans were released from the protein and APTS labeled with GlycanAssure APTS kit (Thermo Fisher) and analyzed on a 3500xL genetic analyzer (Thermo Fisher) capillary electrophoresis instrument. Glycans were assigned based on retention times of known standard glycans as described before (52).

### HIV reservoir size quantification.

Cell-associated (CA)-DNA and CA-RNA of PBMCs were analyzed by real-time PCR as described elsewhere (53). In brief, DNA and RNA was isolated from cryopreserved peripheral blood mononuclear cells (PBMCs) using the AllPrep DNA/RNA minikit (Qiagen, Germany) and conserved regions of HIV long terminal repeat (LTR)/gag primers/probes targeting described before (54).

### Plasma cytokines.

Plasma concentration of epidermal growth factor (EGF), Eotaxin, granulocyte colony-stimulating factor (G-CSF), granulocyte-macrophage colony-stimulating factor (GM-CSF), IFN-α2, IFN-γ, IL-10, IL-12P40, IL-12P70, IL-13, IL-15, IL-17A, IL-1 receptor antagonist (IL-1RA), IL-1α, IL-1β, IL-2, IL-3, IL-4, IL-5, IL-6, IL-7, IL-8, IP-10, MCP-1, MIP-1α, MIP-1β, TNF-α, TNF-β, VEGF, FGF-2, TGF-α, FIT-3L, Fractalkine, GRO, MCP-3, MDC, sCD40L, and IL-9 were analyzed using the MILLIPLEX MAP human cytokine/chemokine magnetic bead panel (premixed 38-plex) (Millipore, MA, USA) according to the manufacturer’s protocol and analyzed on a BioPlex 3D (three-dimensional) suspension array system (Bio-Rad, CA, USA).

### Computational model.

We built a random forest classifier to discriminate fast and slow rebounders using a minimal set of features. The minimal set of features was obtained by recursive feature elimination (function rfe of R package “caret”). The sizes of the compared feature sets were the full set of 62, 32, 16, 8, 4, and 2. Based on the full set of features, the importance of features was calculated as mean decrease in accuracy is determined. For each size, the features with the lowest importance were removed to obtain a set of required size, a random forest model using these features was trained in a fivefold cross-validation framework, and the feature importance was updated. The final set is chosen as the smallest set of features providing the best cross-validation accuracy. The robustness of the modeling approach, including the feature selection and subject classification, was evaluated in a fivefold cross-validation framework, for which the feature selection and subject classification were performed for each fold. This was repeated 20 times to account for different splits of the data. The significance of the overall modeling approach, including the feature selection and subject classification, was evaluated using two control approaches. For each fold, (i) size-matched sets of features were randomly chosen and used to build the classifier, and (ii) permutation testing (55), for which the feature selection and classification were applied to data with randomly shuffled labels (slow/fast). These approaches were repeated 100 times to obtain a distribution of cross-validation accuracies. We calculated *P* values based on the probability that the accuracy of the permuted and random feature model is higher than for the actual model. The feature elimination and classification were performed using the R packages “caret” and “randomForest.” An orthogonal partial least square discriminant analysis (O-PLS-DA) was used to obtain latent variables based on the selected features to visualize the data. The O-PLS-DA model was built using the R package “ropls.”

### Statistics.

If not stated otherwise, boxplots were generated and statistical differences between two groups were calculated by Mann-Whitney test in R (v.3.6.1) and R Studio (v.1.2) and *P* values for adjusted for multiple comparison by Benjamini-Hochberg procedure for systems serology (*n* = 62 parameters), cytokines (*n* = 4 parameters), and reservoir (*n* = 2 parameters) (significance levels, *, *P* < 0.05; **, *P* < 0.01; ***, *P* < 0.001). All experimental data are available in Table S2 in the supplemental material. Principal-component analysis (PCA) was performed in R Studio and visualized with the factoextra package (v.1.0.7). Radar plots were visualized with the fsmb package (v.0.7) in R Studio, and the average of the Z-scored data per variable and group is shown. Linear models were fitted with the stats package (v.4.0.1) and visualized with ggplot2 (v.3.3.2) in R and R Studio.

10.1128/mBio.00170-21.2 TABLE S2 Complete experimental data set for the 23 selected STACCATO study participants. Download Table S2, XLSX file, 0.03 MB. Copyright © 2021 Bartsch et al. 2021 Bartsch et al. https://creativecommons.org/licenses/by/4.0/ This content is distributed under the terms of the Creative Commons Attribution 4.0 International license.

### Study approval.

The STACCATO study is registered at ClinicalTrials.gov (NCT00113126) and was accepted by the local ethics committees from the different participating centers. Written informed consent was obtained from each participant prior study participation. All participants in the RV397 and RV411 studies provided informed consent. The study was approved by the institutional review boards of Chulalongkorn University in Thailand, University of Montreal in Canada, and University of California San Francisco. Secondary use of sample specimens was reviewed and approved by the Partners Health Care Institutional Review Board.
