# Supplementary material for: Viral Rebound Kinetics Correlate with Distinct HIV Antibody Features
Source: mBio. 2021 Mar 9;12(2):e00170-21. doi: 10.1128/mBio.00170-21 (PMC8092214; doi:10.1128/mBio.00170-21)
Supplement: TABLE S1 [file mBio.00170-21-st001.pdf]

Participant-ID	Gender	Age at ATI	Years on ART	VL pre-ART (copies/ml)	CD4 [count] pre-ART (cells/mm^3)	CD4 [count] ATI (cells/mm^3)	VL ATI (copies/ml)
71033	F	28.85	4.64	500	557	525	50
71087	M	21.38	0.98	867	283	355	50
71095	F	37.06	4.1	NA	NA	379	50
71128	F	27.59	2.82	3960	343	536	50
71131	F	36.88	2.84	36977	240	335	50
71136	M	43.87	2.84	59528	220	471	50
71138	M	51.14	2.84	30048	305	691	50
71164	M	33.2	0.59	131000	298	972	50
71180	M	26.8	0.75	120000	308	738	50
72021	F	34.77	0.52	15000	365	407	50
73012	M	53.82	3.07	716000	156	386	50
73014	F	37.35	6.92	NA	441	579	50
73019	F	38.51	1.15	28200	209	720	50
74005	M	27.96	2.34	23074	320	360	50
74008	M	39.25	0.88	NA	132	372	50
77002	M	30.27	0.82	7450	398	453	50
77010	F	42.03	0.54	9640	276	548	50
77018	F	27.79	0.56	6310	239	588	50
77022	F	37.29	3.48	209316	293	565	50
77030	M	37.97	1.46	6800	242	483	50
77046	M	39.93	0.54	179000	252	442	50
77051	F	37.8	2.94	NA	NA	385	50
77054	F	43.88	0.52	63700	388	589	50
