# Supplementary figures and images for: Viral Rebound Kinetics Correlate with Distinct HIV Antibody Features
Source: mBio. 2021 Mar 9;12(2):e00170-21. doi: 10.1128/mBio.00170-21 (PMC8092214; doi:10.1128/mBio.00170-21)

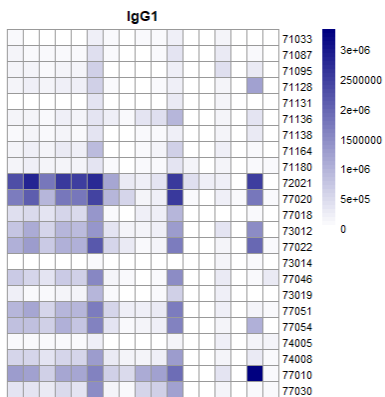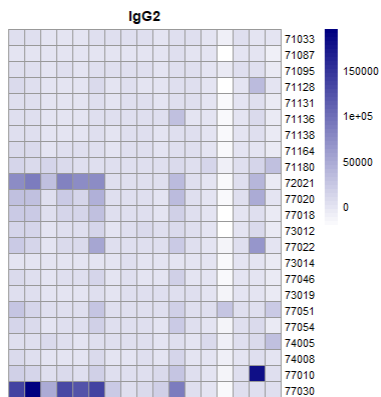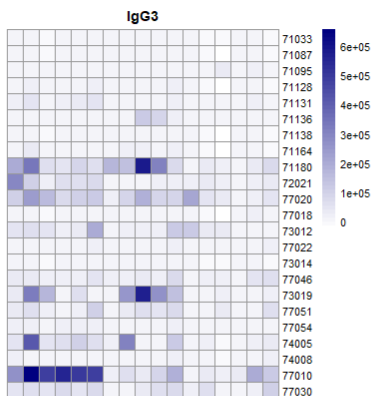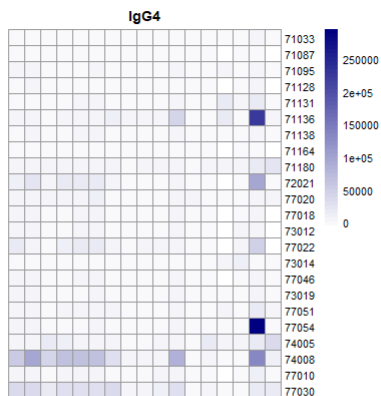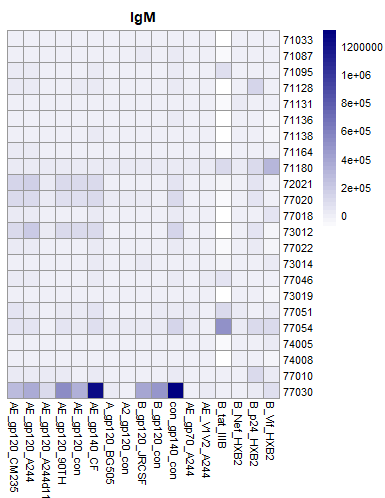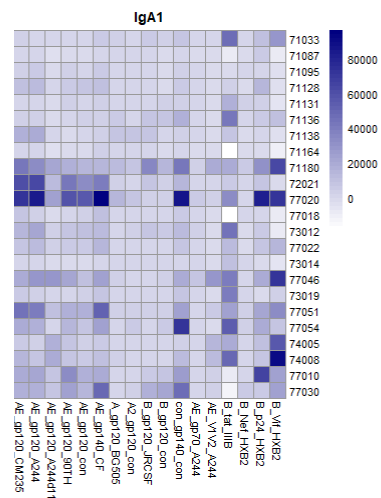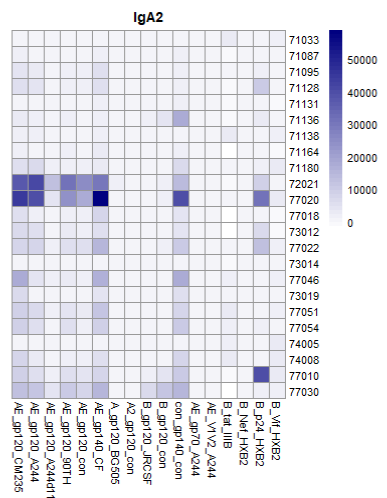

Supplement: FIG S1 [file mBio.00170-21-sf001.pdf]

IgG1

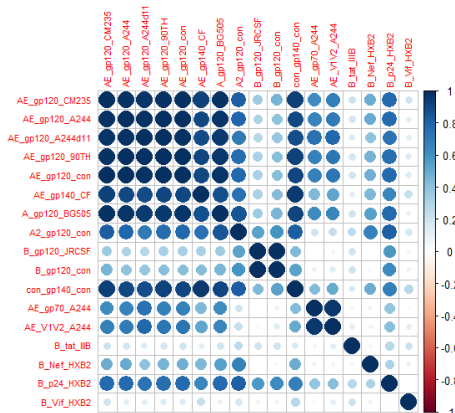

IgG2

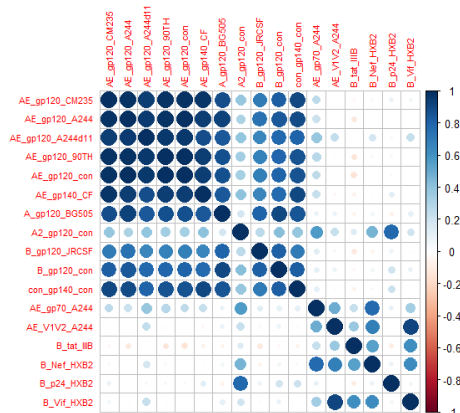

IgG3

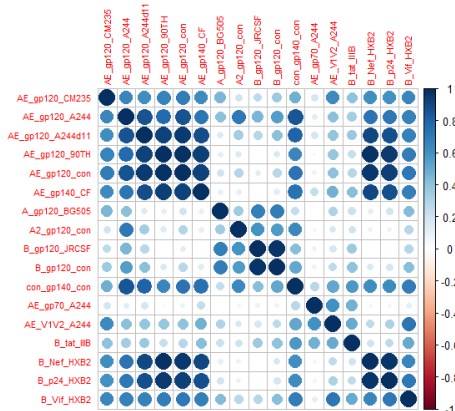

IgG4

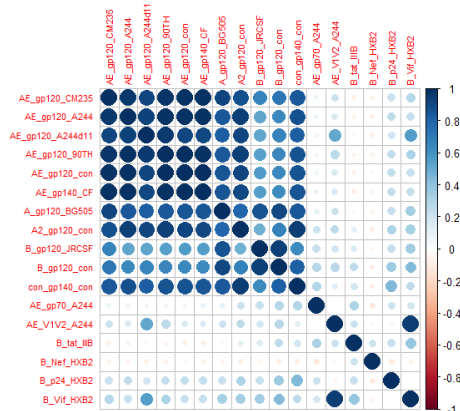

IgA1

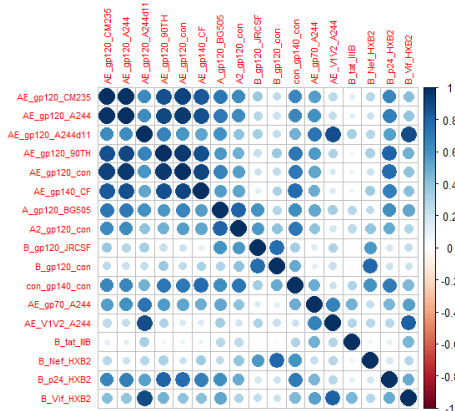

IgA2

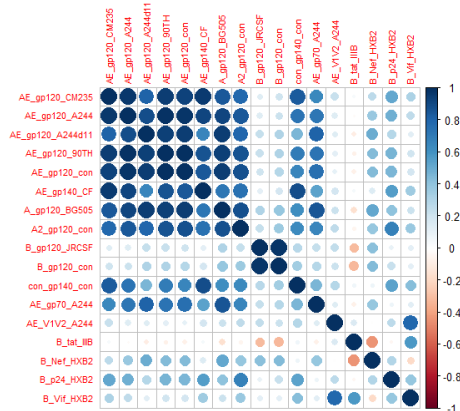

IgM

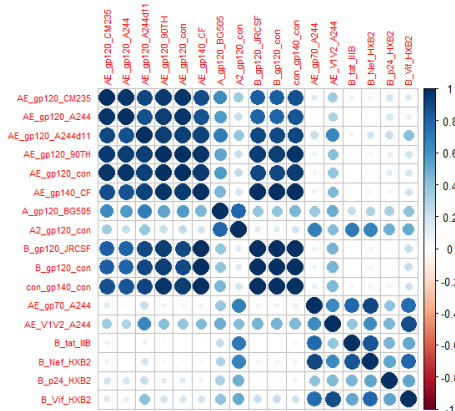

Supplement: FIG S2 [file mBio.00170-21-sf002.pdf]

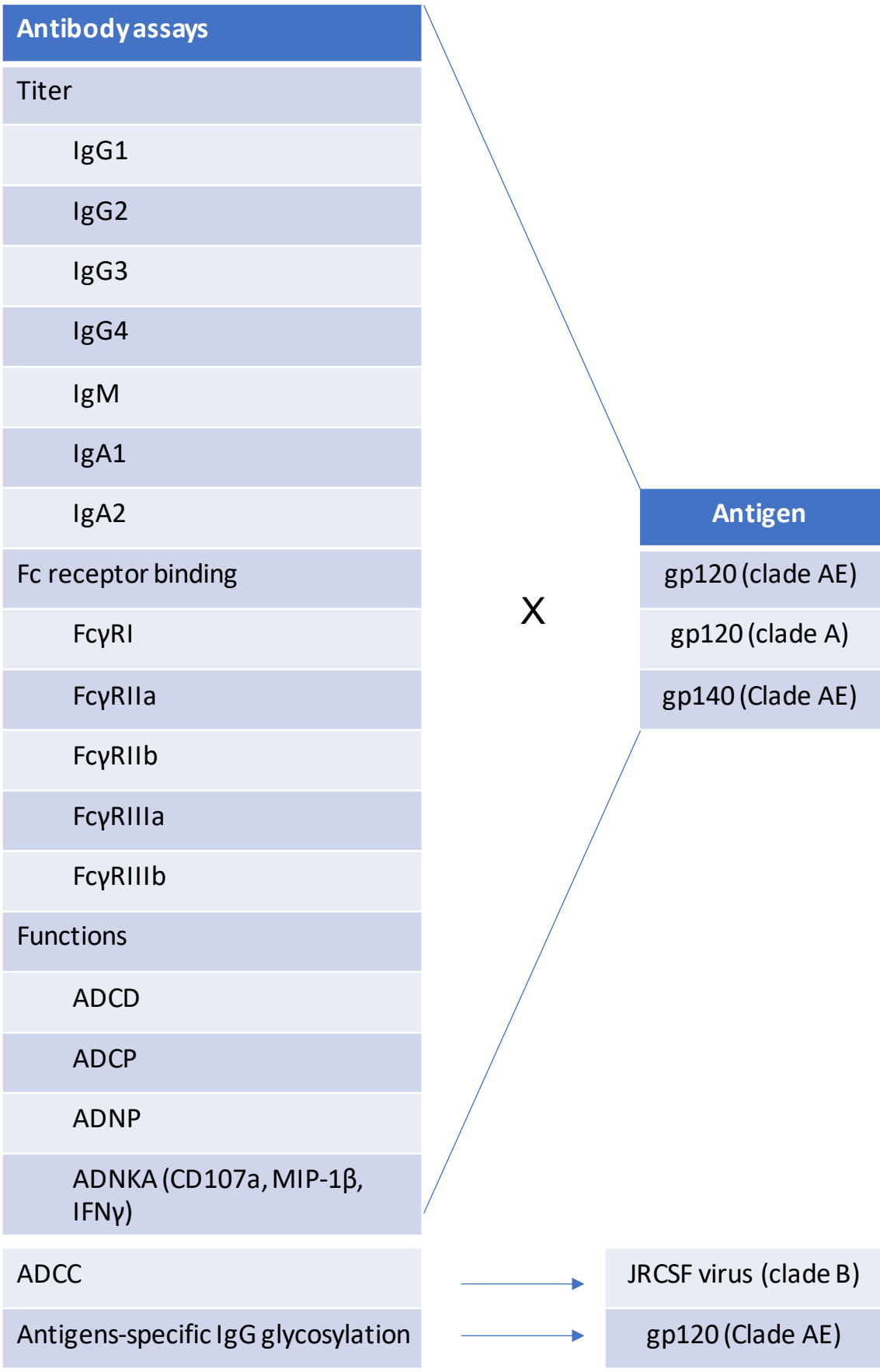

Supplement: FIG S3 [file mBio.00170-21-sf003.pdf]

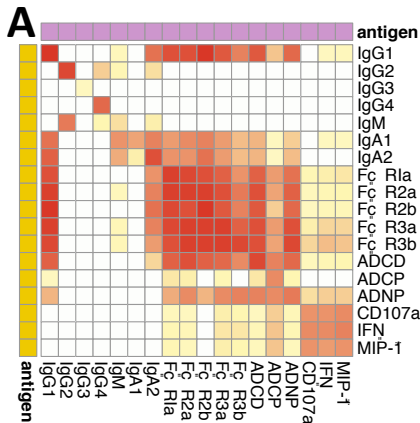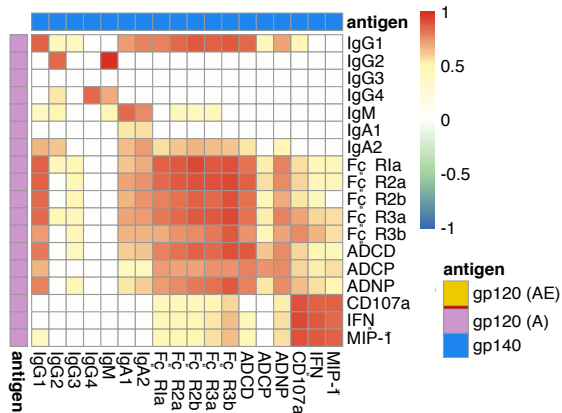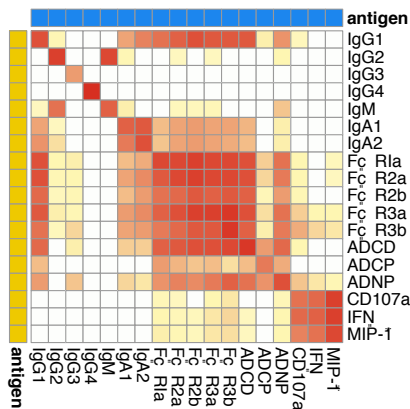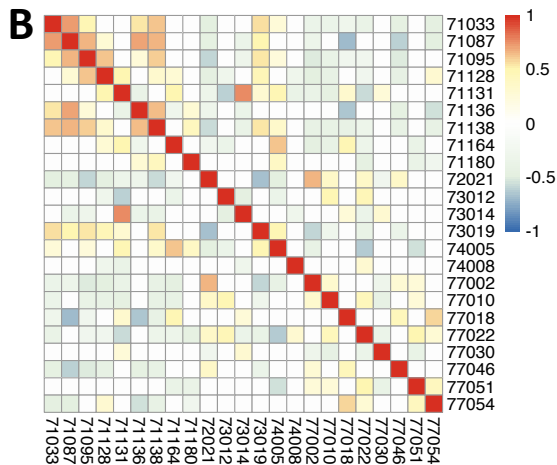

Supplement: FIG S4 [file mBio.00170-21-sf004.pdf]

**A**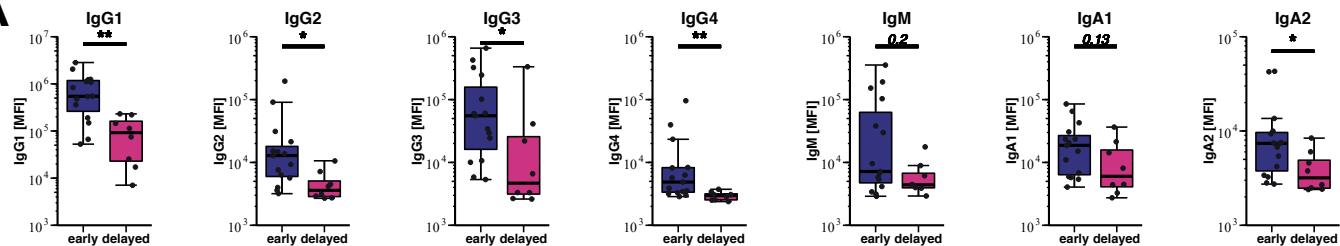**B**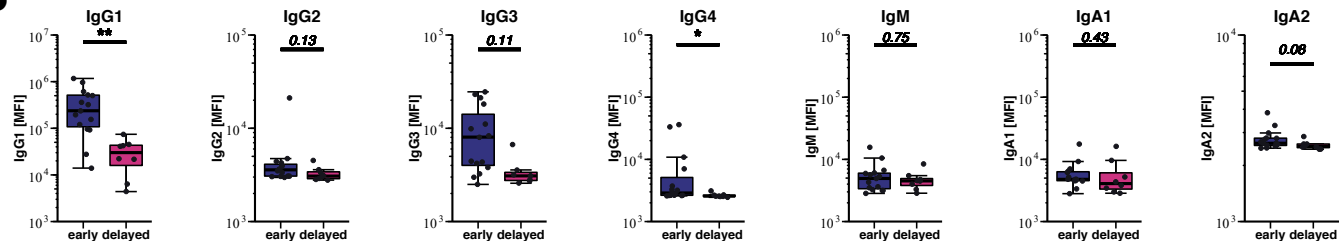**C**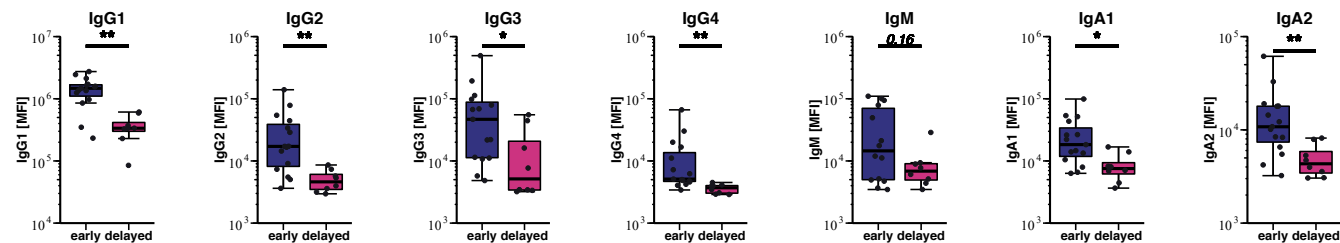

Supplement: FIG S5 [file mBio.00170-21-sf005.pdf]

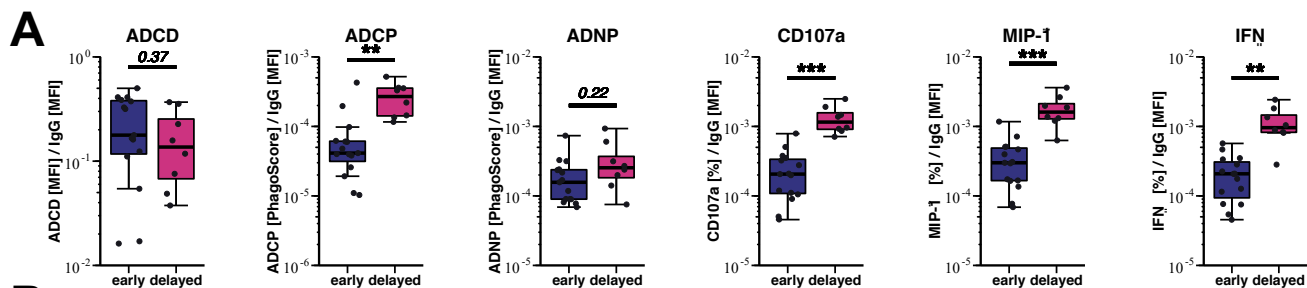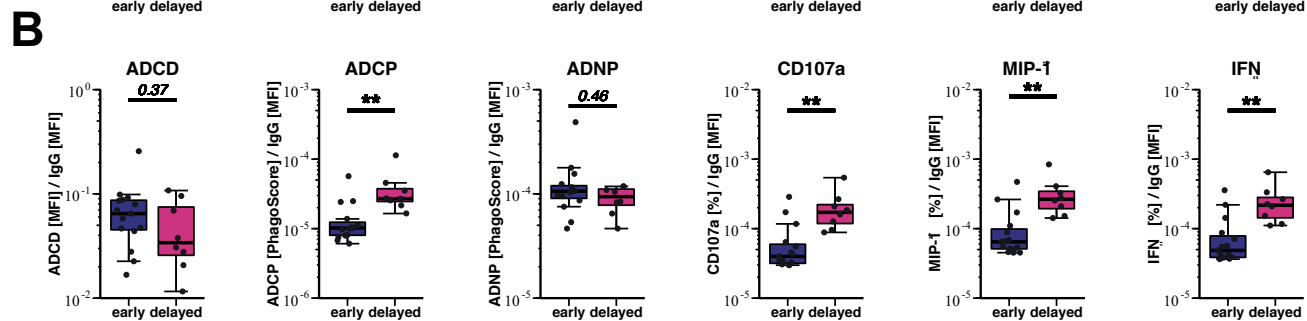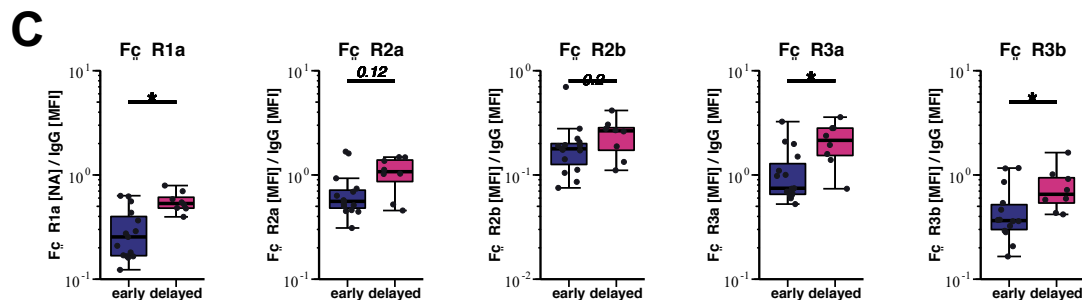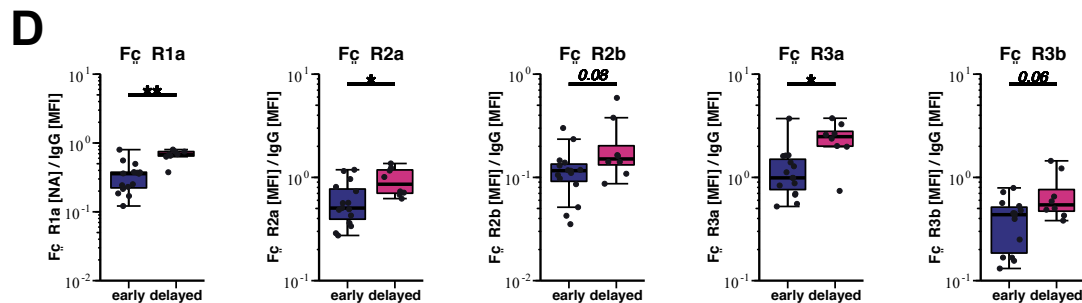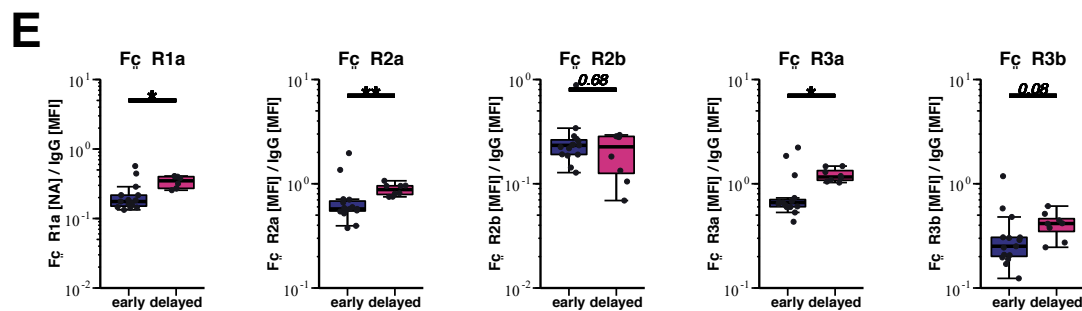

Supplement: FIG S6 [file mBio.00170-21-sf006.pdf]

**A**

ART duration prior to ATI

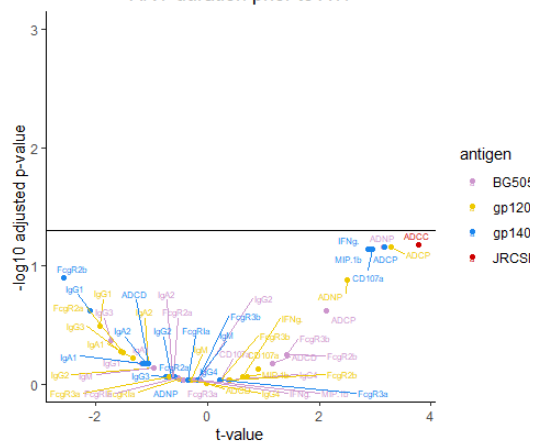**B**

CD4 counts at ATI

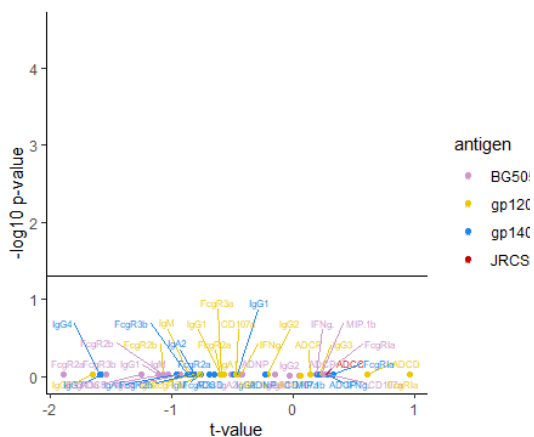**C**

CD4 count prior to ART

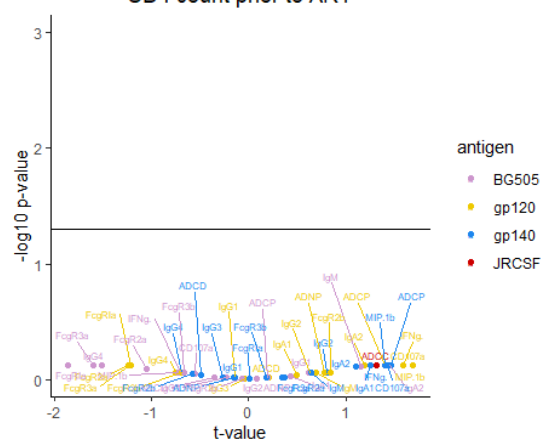**D**

Viral load prior to ART

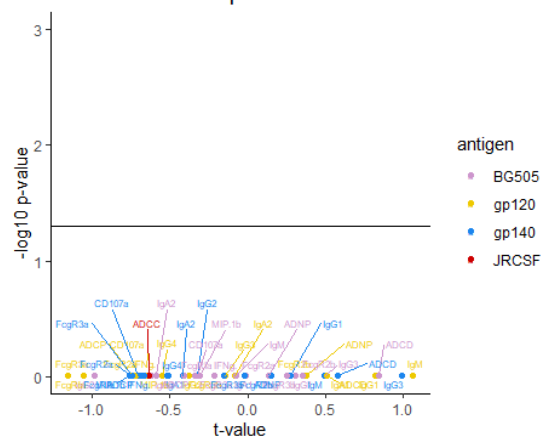

Supplement: FIG S7 [file mBio.00170-21-sf007.pdf]

**A**

gp120 clade AE

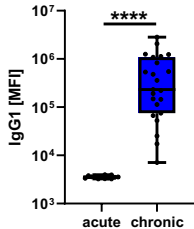

gp120 clade B

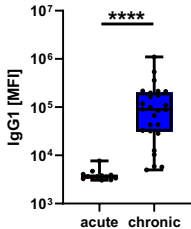**B**

gp120 clade AE

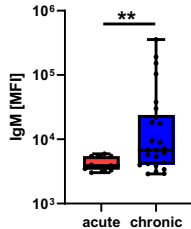

gp120 clade B

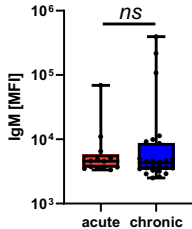

Supplement: FIG S8 [file mBio.00170-21-sf008.pdf]
